# FOXD3-AS1 Knockdown Suppresses Hypoxia-Induced Cardiomyocyte Injury by Increasing Cell Survival and Inhibiting Apoptosis *via* Upregulating Cardioprotective Molecule miR-150-5p *In Vitro*

**DOI:** 10.3389/fphar.2020.01284

**Published:** 2020-08-20

**Authors:** Jiayong Zheng, Bangtian Peng, Yanwei Zhang, Feng Ai, Xiaosong Hu

**Affiliations:** Department of Children’s Heart Center, Henan Province People’s Hospital, Fuwai Central China Cardiovascular Hospital, Zhengzhou, China

**Keywords:** FOXD3-AS1, miR-150-5p, hypoxia, cardiomyocyte, apoptosis, congenital heart disease

## Abstract

Congenital heart disease (CHD) is the most common type of human innate malformation in fetuses. LncRNAs have been pointed to play critical regulatory roles in various types of cardiac development and diseases including CHD. Our study aimed to explore the effects of lncRNA forkhead box D3 antisense RNA 1 (FOXD3-AS1) on hypoxia-induced injury in AC16 cardiomyocytes and the related molecular mechanism. *In vitro* cell model of CHD was established by stimulating AC16 cells with hypoxia (1% O_2_). Expression of FOXD3-AS1 and miR-150-5p was detected by qRT-PCR. Hypoxia-induced injury was evaluated by detecting cell survival, lactate dehydrogenase (LDH) release, apoptosis, and caspase-3/7 activity using MTT, LDH assay, flow cytometry analysis, and caspase-3/7 activity assay, respectively. The regulatory relationship between FOXD3-AS1 and miR-150-5p was explored by luciferase reporter assay, RNA immunoprecipitation (RIP), and qRT-PCR. Results showed that hypoxia exposure caused an upregulation of FOXD3-AS1 and a downregulation of miR-150-5p in AC16 cells. Knockdown of FOXD3-AS1 attenuated reduction of cell survival and increase of LDH release, apoptosis, caspase-3/7 activity, and Bcl-2 associated X (Bax) expression induced by hypoxia in AC16 cells. Notably, we demonstrated that FOXD3-AS1 directly interacted with miR-150-5p to inhibit its expression. miR-150-5p knockdown reinforced the reduction of survival and induction of apoptosis by hypoxia and attenuated the effects of FOXD3-AS1 silencing on the same parameters in AC16 cells. In conclusion, FOXD3-AS1 knockdown protected AC16 cardiomyocytes from hypoxia-induced injury by increasing cell survival and inhibiting apoptosis through upregulating miR-150-5p.

## Introduction

Congenital heart disease (CHD), occurring in approximately 1% of all live births, is believed as one of the most prevalent human innate malformations in fetuses and has become the primary cause of neonatal mortality all over the world (Blue et al., 2012; Cassidy et al., 2015). Recently, research on CHD has evoked considerable interest due to its severe clinical features, such as hypoxia and heart failure (Trojnarska et al., 2009). Several published studies have documented that the pathogenesis of CHD is very complicated, involving various genetic and environmental factors (Jenkins et al., 2007; Varga et al., 2010). Hypoxia is a well-recognized inevitable pathophysiological process associated with CHD, which can affect energy metabolism and therefore lead to heart remodeling (Cordina and Celermajer, 2010). Even though CHD patients are always under the condition of deoxygenated blood perfusion, the majority are still able to survive for a long time and heart failure rarely occurs, indicating that hypoxia may induce compensatory adaptation (Rafiee et al., 2002; Oechslin, 2015). Consequently, an investigation of the underlying adaptive mechanism of cardiomyocytes to hypoxia may be helpful for making improvements in the treatment of CHD.

In recent decades, non-coding RNAs (ncRNAs), accounting for approximately 95% of human genome, are an emerging hotspot of research due to their involvements in the pathogenesis of many cardiac diseases (Kreutzer et al., 2020; Lu and Thum, 2020). microRNAs (miRNAs) is a family of small, highly conserved ncRNAs (~20-22 nucleotides) that serve as post-transcriptional regulators of gene expression by competitively binding to the 3’-untranslated region (3’-UTR) of mRNAs (Rane et al., 2015; Chakraborty et al., 2017; Chakraborty et al., 2020). Importantly, increasing experimental data show that a significantly altered expression of miRNAs is closely implicated in the pathogenesis and development of human cardiovascular diseases (Smith et al., 2015; Mallick et al., 2019; Nagy et al., 2019), and participate in the adaptive process of cardiomyocytes to hypoxia (He et al., 2013).

Long non-coding RNAs (lncRNAs), on the other hand, represent another large group of ncRNA transcripts with over 200 nucleotides in length that have limited or no ability to encode proteins (Jarroux et al., 2017). It has been well acknowledged that lncRNAs function as critical modulators for many aspects of biological and pathological processes, such as cell growth, differentiation, apoptosis, and development (Quinn and Chang, 2016; Hagan et al., 2017). A wide range of documents unveil that lncRNAs play critical regulatory roles in various types of cardiac development and diseases including CHD (Scheuermann and Boyer, 2013). Of note, an increasing number of studies have demonstrated that lncRNA may serve as endogenous sponges or competing endogenous RNAs (ceRNAs) for miRNAs to affect their post-transcriptional regulation and biological functions (Ballantyne et al., 2016). Intriguingly, lncRNA forkhead box D3 antisense RNA 1 (FOXD3-AS1) was reported to be upregulated in oxygen-glucose deprivation and reoxygenation-treated cardiomyocytes (Tong et al., 2019) and was identified to interact with miR-150-5p (Zhang et al., 2017). Accordingly, we supposed whether FOXD3-AS1 could affect hypoxia-induced injury of cardiomyocytes by serving as a molecular sponge of miR-150-5p.

Herein, our study aimed to determine the expression patterns of FOXD3-AS1 and miR-150-5p in hypoxia-cultured cardiomyocytes. The effects of FOXD3-AS1 and miR-150-5p on the survival and apoptosis of hypoxia-cultured cardiomyocytes as well as the possible mechanism involved were also investigated.

## Materials and Methods

### Cell Culture and Hypoxia Treatment

Human cardiomyocyte line AC16 (Davidson et al., 2005) was got from American Type Culture Collection (Manassas, VA, USA) and fostered in Dulbecco’s modified Eagle’s medium (Thermo Fisher Scientific, Inc., Waltham, MA, USA) containing nutrient mixture F-12 (Sigma-Aldrich, St. Louis, MO, USA), 10% fetal bovine serum (Invitrogen, Carlsbad, CA, USA), and 1% penicillin/streptomycin (Sigma-Aldrich). AC16 cells in the hypoxia group were incubated for the indicated time (24, 48, and 72 h) in a hypoxic chamber (Thermo Fisher Scientific, Inc.) filling with a gaseous mixture of 94% N_2_, 5% CO_2_, and 1% O_2_. AC16 cells incubated in a normoxic environment (74% N_2_, 5% CO_2_, and 21% O_2_) at 37°C were served as the normal group.

### Cell Transfection

Two small interfering RNAs (siRNAs) targeting FOXD3-AS1 (si-FOXD3-AS1-1# and si- FOXD3-AS1-2#), miR-150-5p mimics (miR-150-5p), miR-150-5p inhibitor (anti-miR-150-5p), and their negative control oligonucleotides (si-con, miR-con, and anti-miR-con) were all obtained from GenePharma Co., Ltd (Shanghai, China). AC16 cells were incubated until growing to 70-80% confluence and transiently transfected with these oligonucleotides before exposing to hypoxia with Lipofectamine 2000 (Invitrogen) based on the manufacturer’s protocols.

### Quantitative Real Time Polymerase Chain Reaction (qRT-PCR)

Total RNA was extracted from treated AC16 cells using RNAiso Plus (TaKaRa, Otsu, Shiga, Japan). For measurement of miRNA expression, total RNA was reverse transcribed into cDNA using a TaqMan MicroRNA Reverse Transcription Kit (Ambion, Cambridge, MA, USA). qRT-PCR was performed using a TaqMan PCR kit (Thermo Fisher Scientific, Inc.) on a LightCycler 480 system (Roche Ltd., Basel, Switzerland). For detection of mRNA expression, a High-Capacity cDNA Reverse Transcription Kit (TaKaRa) was taken to convert total RNA into cDNA. qRT-PCR was performed using the SYBR Green qPCR Master Mix (TaKaRa) on a LightCycler 480 system (Roche Ltd., Basel, Switzerland). GAPDH and U6 small nuclear RNA (snRNA) were used as the reference housekeeping gene for FOXD3-AS1 and miR-150-5p, respectively. The calculation was carried out according to the 2^-ΔΔCt^ method. The primer sequences were listed as below: FOXD3-AS1, forward: 5′-GGTG GAGG AGGC GAGG ATG-3′, reverse: 5′-AGCG GACA GACA GGGA TTGG-3′; miR-150-5p, forward: 5′-TCGG CGTC TCCC AACC CTTG TAC-3′, reverse: 5′-GTCG TATC CAGT GCAG GGTC CGAG GT-3′.

### 3-(4,5-Dimethylthiazol-2-yl)-2,5-Diphenyl Tetrazolium Bromide (MTT) Assay

MTT assay was adopted to estimate cell survival rate. In brief, AC16 cells were seeded into 96-well plates at a density of 2 × 10^4^ cells per well and transfected with si-FOXD3-AS1, si-con, anti-miR-150-5p, anti-miR-con, si-con + anti-miR-con, si-con + anti-miR-150-5p, si-FOXD3-AS1 + anti-miR-con or si-FOXD3-AS1 + anti-miR-150-5p, followed by incubation under hypoxic condition for 48 h. Afterwards, 20 μl of MTT stock solution (5 mg/ml, Sigma-Aldrich) was supplemented into each well and cultured for 4 h before the formazan crystals was solubilized by adding 150 μl dimethyl sulfoxide (DMSO, Sigma-Aldrich) to each well. Finally, the optical density was recorded at a wavelength of 490 nm with an Infinite M200 microplate spectrophotometer (Tecan, Salzburg, Austria).

### Lactate Dehydrogenase (LDH) Assay

Following different treatments, the culture media were collected and centrifuged to remove cellular debris. LDH release in the culture media was detected using a commercial LDH Kit (Jiancheng Bioengineering Institute, Nanjing, China).

### Western Blotting

Western blotting was performed as previously described (Casasanta et al., 2017). Membrane was incubated with antibodies against Bcl-2 associated X (Bax; Cell Signaling, Danvers, MA, USA) and GAPDH (Cell Signaling), followed by incubation with peroxidase-conjugated secondary antibody (Cell Signaling). The band density was quantified using Quantity One software (Bio-Rad, Hercules, CA, USA) with GAPDH as an internal reference.

### Measurement of Caspase-3/7 Activity

The caspase-3/7 activity in the supernatant of treated AC16 cells was measured by means of a commercially available Caspase-Glo 3/7 assay kit (Promega, Madison, WI, USA) following the manufacturer’s description.

### Apoptosis Assay

The fluorescein isothiocyanate (FITC)-Annexin V/propidium iodide (PI) detection kit (BioVision, Mountain View, CA, USA) was recruited to examine the apoptosis of treated AC16 cells. Following different treatments, AC16 cells were harvested by trypsin digestion and washed twice with ice-cold PBS. After AC16 cells were incubated in 100 μl binding buffer containing 5 μl FITC-Annexin V and 5 μl PI for 15 min in the dark at room temperature, the apoptotic cells was analyzed using FACS Calibur flow cytometry (Becton Dickinson Co, San Jose, CA, USA). The rates of early and late apoptosis were defined as apoptosis rates.

### Luciferase Reporter Assay

The fragments of FOXD3-AS1 containing the wild-type (WT) or mutated (MUT) binding sites of miR-150-5p were amplified with PCR and assembled into the downstream of the luciferase gene in psiCHECK-2 vector (Promega), generating psiCHECK-2-WT-FOXD3-AS1 (termed as WT-FOXD3-AS1) and psiCHECK-2-MUT-FOXD3-AS1 (termed as MUT-FOXD3-AS1). For luciferase reporter assay, AC16 cells were cotransfected with 50 nM miR-con or miR-150-5p and 100 ng of plasmid DNA using Lipofectamine 2000 (Invitrogen). At 48 h post-transfection, the cells were harvested and firefly and Renilla luciferase activities were measured using the Dual-Glo™ Luciferase Assay System (Promega).

### RNA Immunoprecipitation (RIP)

The relationship between FOXD3-AS1 and miR-150-5p was determined by RIP assay using the Magna RIP RNA-binding Protein Immunoprecipitation Kit (Millipore, Billerica, MA, USA). In brief, the lysis of AC16 cells was performed in NP-40 lysis buffer supplemented with protease inhibitor cocktail (Roche Diagnostics, Basel, Switzerland). Thereafter, cell extracts were incubated with RIP buffer containing magnetic beads coupled with anti-human Argonaute 2 (Ago2) (Abcam, Cambridge, MA, USA) or negative control anti-IgG (Abcam) at 4°C overnight. Thereafter, the samples were digested with RNase-free DNase I (Promega) and proteinase K with shaking to remove extra DNAs and proteins. Finally, the immunoprecipitated RNA was isolated and subjected to qRT-PCR analysis for the detection of the relative enrichment of FOXD3-AS1 and miR-150-5p.

### Statistics

Results were shown as mean ± standard deviation (SD) and were analyzed using Graphpad Prism version 5.0 (GraphPad Software, Inc. San Diego, CA, USA). Statistical comparisons between groups were analyzed using Student’s *t* test or one-way analysis of variance (ANOVA). Differences were rendered statistically significant when *P* values were less than 0.05.

## Results

### FOXD3-AS1 Was Upregulated While miR-150-5p Was Downregulated in Hypoxia-Cultured AC16 Cells

**Figure 1:**
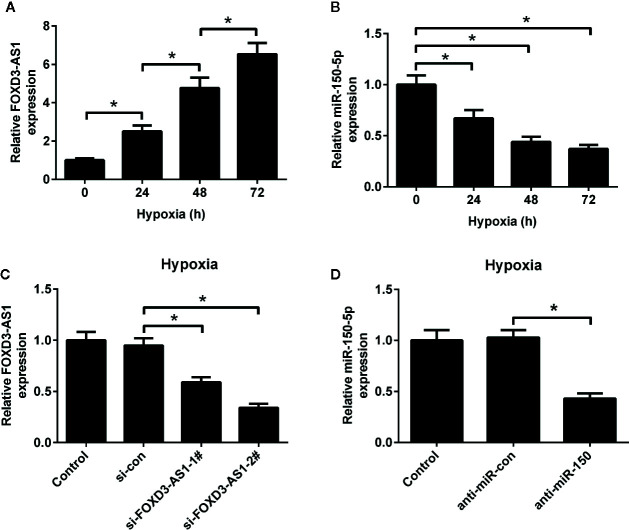
Expression profiles of FOXD3-AS1 and miR-150-5p in hypoxic-cultured AC16 cells. The expression of FOXD3-AS1 **(A)** and miR-150-5p **(B)** was estimated by qRT-PCR after AC16 cells were exposed to hypoxia for 24 h, 48 h, and 72 h. **(C)** qRT-PCR analysis of FOXD3-AS1 expression in AC16 cells after transfection with si-FOXD3-AS1-1#, si-FOXD3-AS1-2# or si-con. **(D)** qRT-PCR analysis of miR-150-5p expression in AC16 cells delivered with anti-miR-150-5p or anti-miR-con. *P < 0.05. All experiments were repeated three times.

The expression patterns of FOXD3-AS1 and miR-150-5p in hypoxic AC16 cells were detected by qRT-PCR. As shown in **Figures 1A, B**, the abundance of FOXD3-AS1 was elevated and miR-150-5p expression was reduced to some extent in AC16 cells in a time-dependent manner. To characterize the functional roles of FOXD3-AS1 and miR-150-5p in hypoxia-treated AC16 cells, FOXD3-AS1 expression was decreased in AC16 cells by transfecting with si-FOXD3-AS1-1# or si-FOXD3-AS1-2# (**Figure 1C**) and miR-150-5p expression was inhibited by transfection with anti-miR-150-5p (**Figure 1D**). Si-FOXD3-AS1-2# (hereafter referred to as si-FOXD3-AS1) with higher knockdown efficiency was used in the subsequent assays.

### Knockdown of FOXD3-AS1 Increased the Survival of Hypoxia-Cultured AC16 Cells

**Figure 2:**
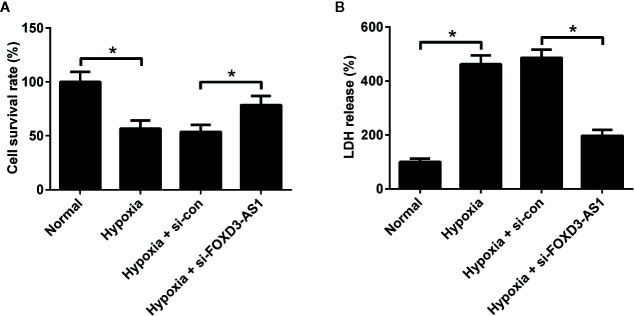
Effects of FOXD3-AS1 knockdown on the survival of hypoxia-treated AC16 cells. AC16 cells were transfected with si-FOXD3-AS1 or si-con prior to treatment with hypoxia for 48 h, followed by evaluation of cell survival **(A)** and lactate dehydrogenase (LDH) release **(B)** by MTT assay and LDH assay, respectively. *P < 0.05. All experiments were conducted in triplicate and repeated three times.

To clarify the effect of FOXD3-AS1 on the survival of hypoxic AC16 cells, MTT assay was carried out. The results revealed that hypoxia stimulation led to a substantial decline of cell survival rate in AC16 cells, while FOXD3-AS1 silencing ameliorated hypoxia-induced survival reduction (**Figure 2A**). Meanwhile, cell injury was determined by measuring the LDH release. LDH assay showed that depletion of FOXD3-AS1 attenuated hypoxia-induced LDH release of AC16 cells (**Figure 2B**). These results suggested that knockdown of FOXD3-AS1 promoted the survival of hypoxia-cultured AC16 cells.

### Knockdown of FOXD3-AS1 Inhibited Hypoxia-Induced AC16 Cell Apoptosis

**Figure 3:**
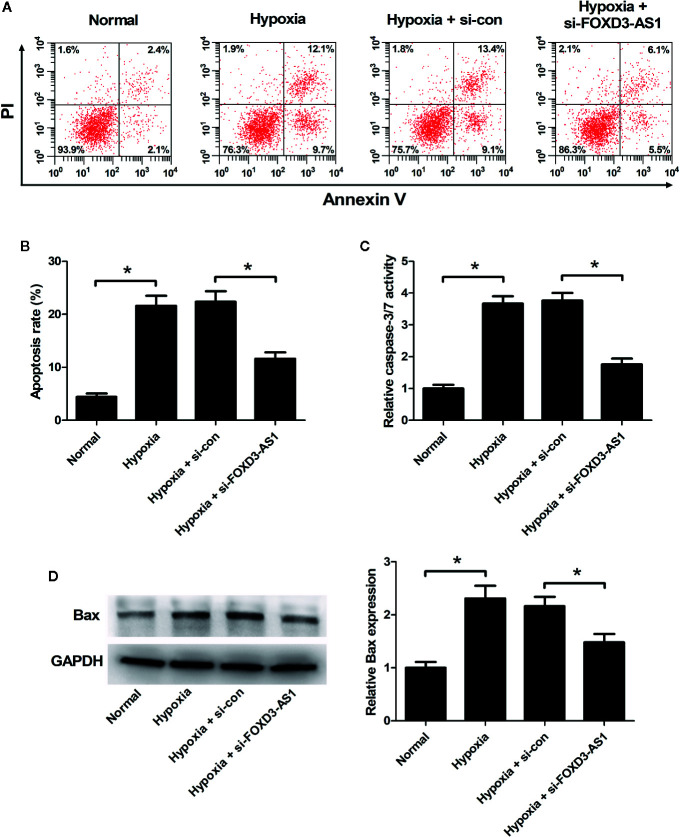
Effects of FOXD3-AS1 knockdown on the apoptosis of hypoxia-treated AC16 cells. AC16 cells were transfected with si-FOXD3-AS1 or si-con and then exposed to hypoxia for 48 h, followed by the determination of apoptosis **(A, B)**, caspase-3/7 activity **(C)**, and Bax expression **(D)** by flow cytometry analysis, caspase-3/7 activity assay, and western blotting, respectively. *P < 0.05. All experiments were repeated three times.

The effect of FOXD3-AS1 on the apoptosis of AC16 cells in the context of hypoxia was explored using flow cytometry analysis and caspase-3/7 activity assay. As displayed in **Figures 3A–C**, the induction of hypoxia caused a higher level of cell apoptosis and increased caspase-3/7 activity in AC16 cells, while these effects were antagonized in response to the knockdown of FOXD3-AS1. Hypoxia exposure increased pro-apoptotic protein Bax expression, but knockdown of FOXD3-AS1 alleviated this effect (**Figure 3D**). Together, we concluded that knockdown of FOXD3-AS1 inhibited hypoxia-induced AC16 cell apoptosis.

### miR-150-5p Was a Direct Target of FOXD3-AS1

**Figure 4:**
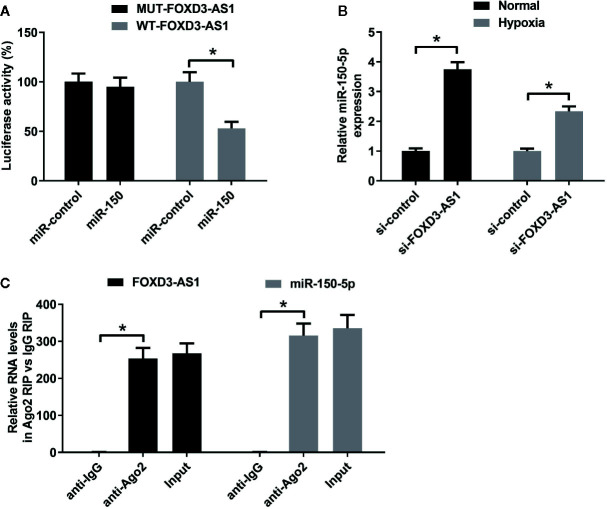
The regulatory relationship between FOXD3-AS1 and miR-150-5p in AC16 cells. **(A)** Measurement of luciferase activity. The constructed luciferase reporter plasmids were transfected into AC16 cells together with miR-150-5p or miR-con and luciferase reporter assay was applied to detect luciferase activity. **(B)** qRT-PCR was conducted to detect miR-150-5p expression after AC16 cells transfected with si-FOXD3-AS1 or si-con were subjected to normoxic or hypoxia treatment for 48 h. **(C)** RIP assay was performed to show the interaction between FOXD3-AS1 and miR-150-5p in AC16 cells and the immunoprecipitated RNA was analyzed by qRT-PCR. *P < 0.05. All experiments were repeated three times.

A previous study has been shown that FOXD3-AS1 contained binding sites for miR-150-5p (Zhang et al., 2017). To confirm it, luciferase reporter assay was carried out using AC16 cells co-transfected with luciferase-reporter plasmids WT-FOXD3-AS1 or MUT-FOXD3-AS1 and miR-150-5p minics. At 48 h post-transfection, luciferase activities were measured. The results uncovered that overexpression of miR-150-5p led to a decrease of luciferase activity in WT-FOXD3-AS1 group, but did not affect the luciferase activity in MUT-FOXD3-AS1 group, confirming the interactions between FOXD3-AS1 and miR-150-5p. (**Figure 4A**). To observe the regulatory influence of FOXD3-AS1 on miR-150-5p expression, AC16 cells were transfected with si-FOXD3-AS1 or si-con, followed by the examination of miR-150-5p expression by qRT-PCR. The results proved that miR-150-5p expression was elevated following silencing of FOXD3-AS1 in AC16 cells under both normal and hypoxia conditions (**Figure 4B**). To show that FOXD3-AS1 and miR-150-5p were both recruited by Ago2 in miRNAs complexes, RIP assay was performed. Results manifested that FOXD3-AS1 and miR-150-5p were both preferentially enriched among Ago2 immunoprecipitates in AC16 cell lysates (**Figure 4C**). These data indicated that FOXD3-AS1 functioned as a miR-150-5p sponge in AC16 cells.

### miR-150-5p Knockdown Attenuated the Effects of FOXD3-AS1 Knockdown on Survival of Hypoxia-Cultured AC16 Cells

**Figure 5:**
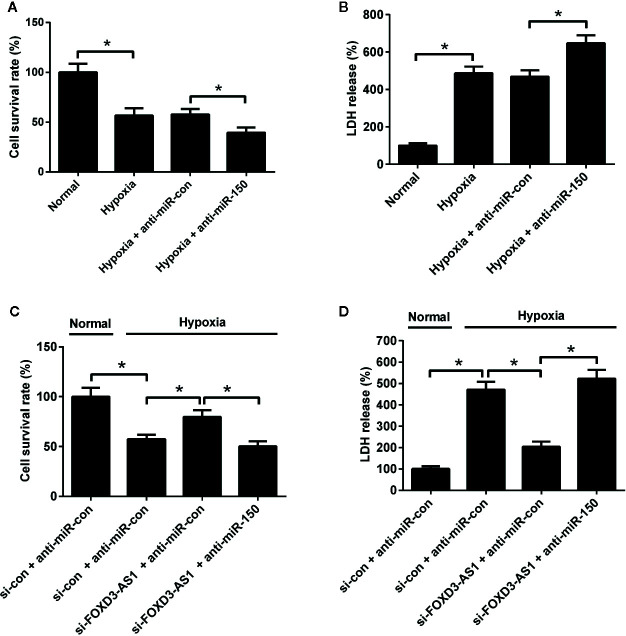
miR-150-5p knockdown abolished the effect of FOXD3-AS1 knockdown on survival of hypoxia-cultured AC16 cells. AC16 cells were transfected with anti-miR-150-5p or anti-miR-con before exposing to hypoxia for 48 h, followed by assessment of cell survival **(A)** and lactate dehydrogenase (LDH) release **(B)** by MTT assay and LDH assay, respectively. AC16 cells were transfected with si-con + anti-miR-con, si-con + anti-miR-150-5p, si-FOXD3-AS1 + anti-miR-con, or si-FOXD3-AS1 + anti-miR-150-5p and incubated under hypoxic condition for 48 h, followed by the evaluation of cell survival **(C)** and LDH release **(D)** by MTT assay and LDH assay, respectively. *P < 0.05. All experiments were conducted in triplicate and repeated three times.

The effects of miR-150-5p on hypoxia-treated AC16 cells were further explored. MTT assay presented that miR-150-5p knockdown reinforced the reduction of survival induced by hypoxia in AC16 cells compared with control group (**Figure 5A**). miR-150-5p knockdown abolished the increase of the survival due to FOXD3-AS1 downregulation in hypoxia-cultured AC16 cells (**Figure 5C**). Meanwhile, LDH assay revealed that transfection with anti-miR-150-5p enhanced hypoxia-induced injury of AC16 cells when compared to anti-miR-con-transfected group (**Figure 5B**). Moreover, we found that the inhibitory effect of FOXD3-AS1 silencing on hypoxia-induced injury on AC16 cells was overturned following reintroduction with anti-miR-150-5p (**Figure 5D**). These results revealed that miR-150-5p knockdown abolished the effects of FOXD3-AS1 knockdown on survival of hypoxia-cultured AC16 cells.

### miR-150-5p Knockdown Resisted the Effect of FOXD3-AS1 on Apoptosis in Hypoxia-Cultured AC16 Cells

**Figure 6:**
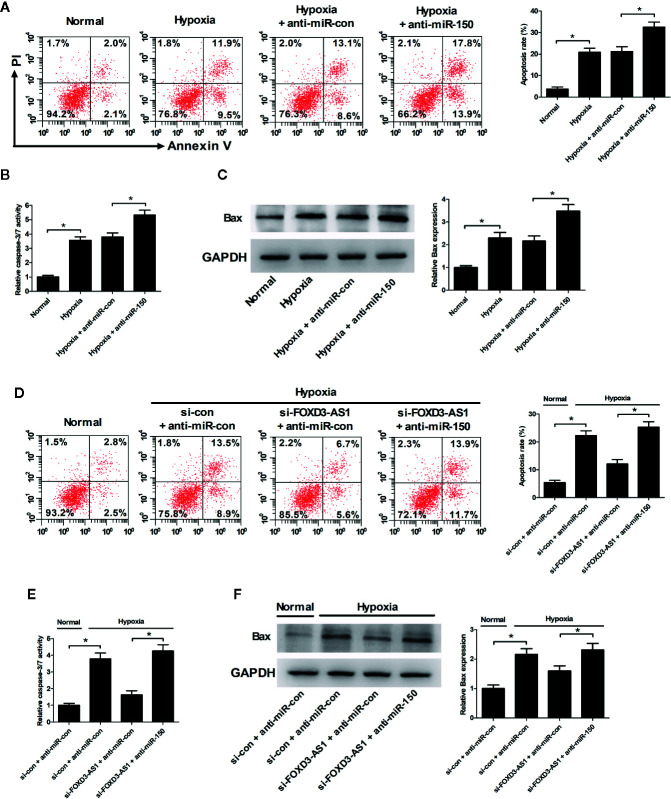
miR-150-5p knockdown rescued the suppressive effect of FOXD3-AS1 silencing on apoptosis in hypoxia-cultured AC16 cells. AC16 cells were transfected with anti-miR-150-5p or anti-miR-con and then exposed to hypoxia for 48 h, followed by the determination of apoptosis **(A)**, caspase-3/7 activity **(B)**, and Bax expression **(C)** by flow cytometry analysis, caspase-3/7 activity assay, and western blotting, respectively. AC16 cells were transfected with si-con + anti-miR-con, si-con + anti-miR-150-5p, si-FOXD3-AS1 + anti-miR-con, or si-FOXD3-AS1 + anti-miR-150-5p and incubated under hypoxic conditions for 48 h, followed by the measurement of apoptosis **(D)**, caspase-3/7 activity **(E)**, and Bax expression **(F)** by flow cytometry analysis, caspase-3/7 activity assay, and western blotting, respectively. *P < 0.05. All experiments were repeated three times.

Flow cytometry analysis and caspase-3/7 activity assay hinted that miR-150-5p inhibition led to a significant enhancement of hypoxia-induced apoptosis (**Figure 6A**) and increase of caspase-3/7 activity and Bax expression (**Figures 6B, C**) in AC16 cells. Rescue experiments demonstrated that interference with anti-miR-150-5p relieved the inhibitory effect of FOXD3-AS1 knockdown on hypoxia-induced apoptosis (**Figure 6D**) and increase of caspase-3/7 activity and Bax expression (**Figures 6E, F**) in AC16 cells. Collectively, these data suggested that miR-150-5p knockdown rescued the effect of FOXD3-AS1 silencing on apoptosis in hypoxia-cultured AC16 cells.

## Discussion

Mounting evidence shows that CHD patients always undergo stresses induced by decreased oxygenation and an increase of overload caused by versatile cardiac defects, thus leading to high rates of morbidity and mortality (He et al., 2013). Hypoxia is commonly observed in patients with CHD and contributes to the pathogenesis of CHD in a complex pattern (Sommer et al., 2008). It has been shown that hypoxia induces injury of AC16 cardiomyocytes by suppressing cell viability and increasing cell apoptosis (Cai and Li, 2019). It is meaningful to figure out endogenous cellular protective mechanisms against hypoxia-induced injury in cardiomyocytes. To the best of knowledge, the present study is the first time to report that FOXD3-AS1 was upregulated and miR-150-5p was downregulated by hypoxia in AC16 cells in a time-dependent manner. FOXD3-AS1 knockdown protected AC16 cells from hypoxia-induced injury by increasing cell survival and inhibiting apoptosis through upregulating miR-150-5p.

A growing number of studies have documented the regulating effects of lncRNAs in the initiation and development of various cardiovascular diseases (Uchida and Dimmeler, 2015). For instance, lncRNA NEAT1 was reported to be upregulated in the ischemia/reperfusion myocardium and knockdown of lncRNA NEAT1 served a protective role against hypoxia/reoxygenation- induced cardiomyocyte injury (Wu et al., 2019). LncRNA ANRIL was demonstrated to be enhanced in hypoxia-induced H9c2 cells and play a protective part in hypoxia-induced H9c2 cell injury in acute myocardial infarction (Shu et al., 2020). LncRNA ROR was revealed to protect H9c2 cells against hypoxia-induced damages in CHD by rescuing viability, suppressing apoptosis and blocking Cytochrome c release (Wang and Yuan, 2019). FOXD3-AS1, located at the chromosome 1p31.3 with 547 bp in length, is the antisense partner of protein coding gene FOXD3. Recently, increasing researches have focused on the involvement of FOXD3-AS1 in multiple human diseases. Chen et al. demonstrated that inhibition of FOXD3-AS1 suppressed the aggressive biological behaviors of thyroid cancer *via* elevating miR-296-5p and inactivating the TGF-β1/Smads signaling pathway (Chen et al., 2019). Tong et al. revealed that FOXD3-AS1 expression was increased in oxygen-glucose deprivation and reoxygenation (OGD/R)-treated H9c2 cells and overexpression of FOXD3-AS1 aggravated ischemia/reperfusion injury of cardiomyocytes through promoting autophagy (Tong et al., 2019). Consistently, our study provided the evidence that hypoxia caused a time-dependent upregulation of FOXD3-AS1 in AC16 cells. Functional experiments further proved that FOXD3-AS1 knockdown significantly attenuated hypoxia-induced survival inhibition and apoptosis in AC16 cells. These results suggested that hypoxia-induced upregulation of FOXD3-AS1 might be a protective mechanism against hypoxia-induced injury of cardiomyocytes. It is worth noting that Zhang et al. revealed that FOXD3-AS1 was upregulated in mouse lung tissues after exposure to hyperoxia and exaggerated hyperoxia-induced lung epithelial cell death (Zhang et al., 2017). This contradiction may result from different cell types.

Considerable evidence has shown that numerous lncRNAs participate in the regulation of the development of cardiovascular diseases through the ceRNA mechanism, in which lncRNAs can act as ceRNAs to bind miRNAs through sponge-like action, therefore modulating their expression and functions (Wang et al., 2014; Song et al., 2016). To make out the molecular mechanism underlying the effects of FOXD3-AS1 knockdown on hypoxia-treated AC16 cells, GenBank database was used to confirm that FOXD3-AS1 contained a potential binding site of miR-150-5p. The subsequent luciferase reporter assay, RIP assay, and qRT-PCR proved that FOXD3-AS1 directly interacted with miR-150-5p to negatively regulate miR-150-5p expression in AC16 cells. Similarly, FOXD3-AS1 was previously demonstrated to serve as a sponge or ceRNA for miR-150-5p in lung hypoxic injury (Zhang et al., 2017). miR-150-5p, a member of the miR-150 family located on human chromosome 19q13, was initially regarded as the main miRNA in immune and haematopoietic cells (He et al., 2014). Studies have demonstrated that miR-150 expression was inhibited in human cardiomyocytes after hypoxia treatment and overexpressing miR-150 protected cardiomyocytes from hypoxia-induced death and apoptosis by targeting glucose-regulated protein-94 (Ma et al., 2018), C-reactive protein (Wu et al., 2017), Signal transducer and activator of transcription 1 and c-Fos (Zhu et al., 2017). In line with the results of these previous studies, we proved that miR-150-5p expression was time-dependently downregulated after exposure to hypoxia in AC16 cells, and miR-150-5p knockdown significantly reinforced hypoxia-induced survival reduction and apoptosis in AC16 cells. Rescue experiments further manifested that miR-150-5p knockdown abolished the effects of FOXD3-AS1 silencing on hypoxia-induced survival reduction and apoptosis in AC16 cells, suggesting that FOXD3-AS1 silencing suppressed hypoxia-induced injury of AC16 cells by upregulating miR-150-5p. Interestingly, a recent paper suggested that miR-150-5p targeted Bax to inhibit hypoxia-induced apoptosis of H9c2 cells (Zhou et al., 2020). In our study, we also found that FOXD3-AS1 knockdown decreased hypoxia-induced Bax expression, and this effect was attenuated by miR-150-5p downregulation. Thus we deduced that FOXD3-AS1 knockdown suppressed hypoxia-induced cardiomyocyte injury by inhibiting Bax expression *via* upregulating cardioprotective molecule miR-150-5p.

**Figure 7:**
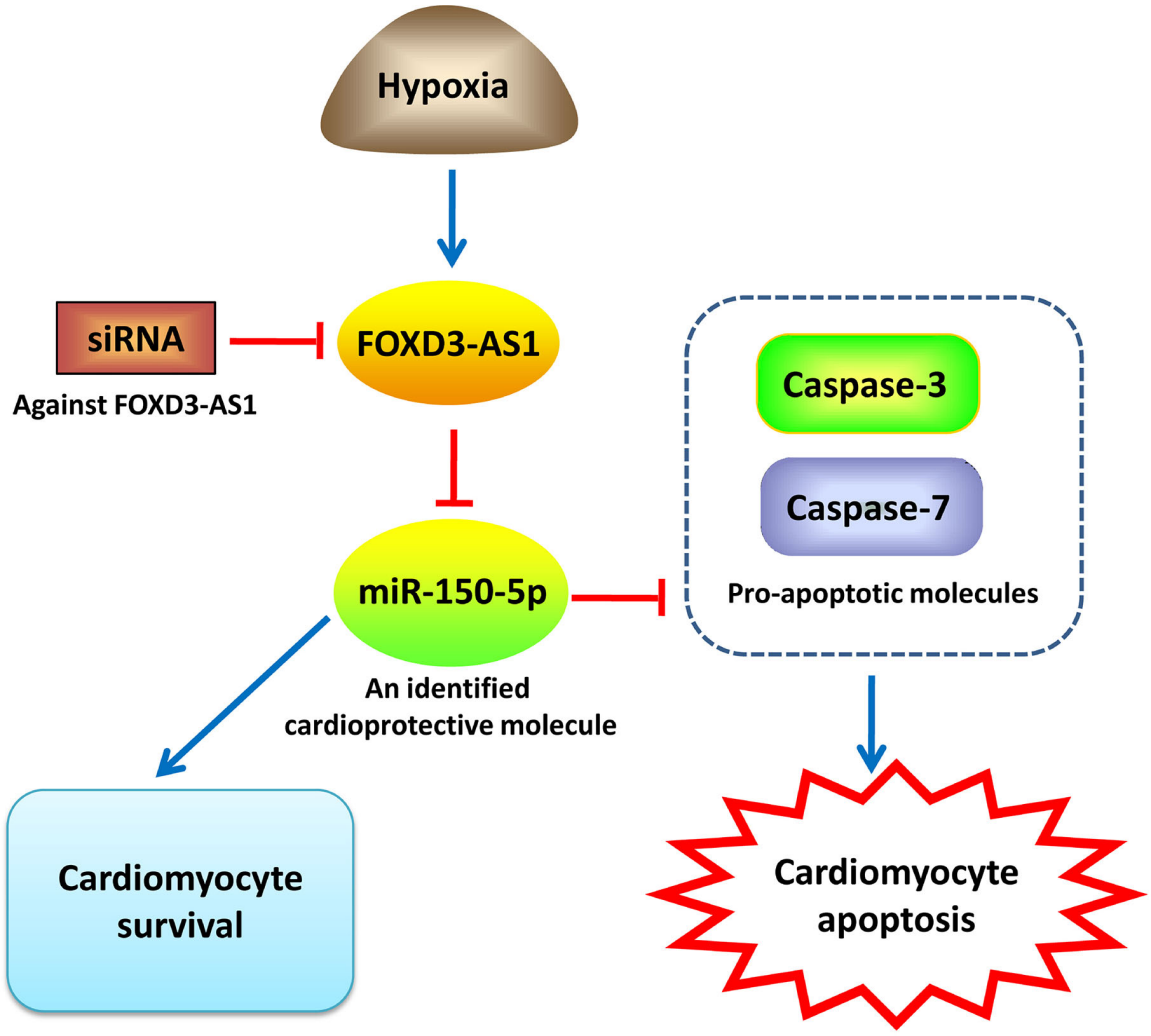
An illustration showing that FOXD3-AS1 knockdown protects AC16 cardiomyocytes from hypoxia-induced injury by promoting cell survival and inhibiting apoptosis through upregulating miR-150-5p.

In summary, our study demonstrated that hypoxia caused an upregulation of FOXD3-AS1 and a downregulation of miR-150-5p in AC16 cells. Depletion of FOXD3-AS1 protected AC16 cardiomyocytes from hypoxia-induced injury by increasing cell survival and inhibiting apoptosis through upregulating miR-150-5p (**Figure 7**). Our study provided a novel promising target and therapeutic direction for the treatment of CHD and other hypoxic heart disease. However, more researches, importantly *in vivo* experiments, are still required to be performed in the future research to confirm this conclusion.

## Data Availability Statement

The original contributions presented in the study are included in the article/supplementary material; further inquiries can be directed to the corresponding author.

## Author Contributions

JZ participated in the conception and design of the study. BP performed the analysis and interpretation of data. YZ contributed to drafting the manuscript. FA and XH reviewed and approved the final submitted manuscript.

## Conflict of Interest

The authors declare that the research was conducted in the absence of any commercial or financial relationships that could be construed as a potential conflict of interest.
